# Visualizing rotation and reversal of the Néel vector through antiferromagnetic trichroism

**DOI:** 10.1038/s41467-022-28215-w

**Published:** 2022-02-04

**Authors:** Kenta Kimura, Yutaro Otake, Tsuyoshi Kimura

**Affiliations:** 1 Department of Advanced Materials Science, University of Tokyo, Kashiwa, 277-8561 Japan; 2 Department of Applied Physics, University of Tokyo, Hongo, 7-3-1, Bunkyo, Tokyo, 113-8656 Japan

**Keywords:** Magnetic properties and materials, Spintronics

## Abstract

Conventional magnetic memories rely on bistable magnetic states, such as the up and down magnetization states in ferromagnets. Increasing the number of stable magnetic states in each cell, preferably composed of antiferromagnets without stray fields, promises to achieve higher-capacity memories. Thus far, such multi-stable antiferromagnetic states have been extensively studied in conducting systems. Here, we report on a striking optical response in the magnetoelectric collinear antiferromagnet Bi_2_CuO_4_, which is an insulating version of the representative spintronic material, CuMnAs, with four stable Néel vector orientations. We find that, due to a magnetoelectric effect in a visible range, which is enhanced by a peculiar local environment of Cu ions, absorption coefficient takes three discrete values depending on an angle between the propagation vector of light and the Néel vector—a phenomenon that we term antiferromagnetic trichroism. Furthermore, using this antiferromagnetic trichroism, we successfully visualize field-driven reversal and rotation of the Néel vector.

## Abstract (web summary)

Antiferromagnets have great promise for use in spin-based electronics; however, detecting the Neel vector is challenging due to the lack of a net magnetization. Here, Kimura et al demonstrate an intriguing optical response, where the optical absorption depends on the angle of the Neel vector.

## Introduction

Antiferromagnetism is defined as ordered magnetism in which the net magnetization is canceled out because of microscopic spin arrangements. Its excellent features suitable for spintronics applications include the robustness against perturbations, no stray fields, and ultrafast dynamics^1,2^. Because of the zero net magnetization, antiferromagnets were originally considered less useful as an active component in spintronic devices. This situation has been drastically changed by recent breakthroughs in the electrical control and detection of the orientation of the Néel vector in metallic collinear antiferromagnets such as CuMnAs and Mn_2_Au^3,4^. These materials possess a tetragonal crystal structure, and the in-plane colinear antiferromagnetic (AFM) ordering yields four energetically stable AFM domains, corresponding to in-plane 90° rotations of the Néel vector. In addition, although their crystal structure is centrosymmetric, local inversion symmetry at magnetic Mn-ion sites is broken. Consequently, the AFM ordering breaks both the parity (P) and time-reversal (T) symmetries, thus allowing for a current-induced staggered torque, which is responsible for both 90° and 180° Néel vector switching^3–8^. It has also been shown that the P- and T-symmetry breaking supports a full detection of four Néel vector orientations^4,7^. Thus, tetragonal in-plane colinear antiferromagnets without local inversion symmetry are promising for the development of multistable magnetic memories.

Besides CuMnAs and Mn_2_Au, multistable memory functionalities have been extensively explored and demonstrated in various antiferromagnets^9–12^. These systems are conductive, and the electrical current accompanying Joule heating is typically used to control the domains. In AFM insulators, by contrast, an electric field without Joule heating is available as an option for direct domain control. A well-known control mechanism is the linear magnetoelectric (ME) effect, which is allowed in a system without P and T symmetries^13,14^. Furthermore, the ME effect in the optical regime^15–27^, often called the optical magnetoelectric (OME) effect, can induce peculiar symmetry-dependent optical responses such as nonreciprocal directional dichroism (NDD), that is, a difference in the absorption coefficient (*A*) between two counter-propagating light beams. Using the OME effect, AFM domains can be optically identified even in fully compensated AFM materials^16,24,26^. Moreover, spatially resolved visualization of AFM domains via the OME effects has been experimentally demonstrated very recently^27^. To date, however, there have been only a few reports on AFM materials exhibiting large OME responses, none of which have multistable (three or more) AFM domains.

**Fig. 1:**
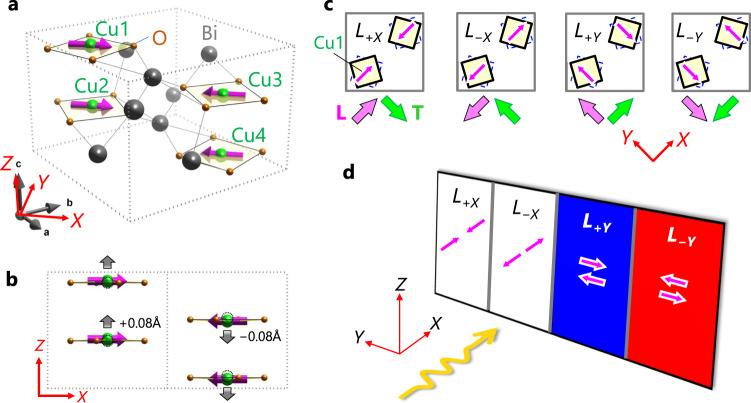
Crystal and magnetic structures of Bi_2_CuO_4_ and conceptual illustration of antiferromagnetic trichroism. **a, b** Three-dimensional (**a**) and *Y*-axis (**b**) views of crystal and magnetic structures of Bi_2_CuO_4_. The [110], $[\bar{1}10]$, and [001] axes are referred to as *X*, *Y*, and *Z*, respectively. Green, brown, and gray balls represent Cu, O, and Bi atoms, respectively. A unit cell (gray dotted lines) contains four Cu ions labeled as Cu1–Cu4. Square-planar CuO_4_ units stack in a twisted manner along the *Z* axis. Magenta arrows denote Cu spins, which are ferromagnetically aligned along the *Z* axis while antiferromagnetically aligned in the *XY* plane. A small displacement (0.08 Å) of a Cu atom from the plane of O_4_ square along the *Z* axis is denoted by gray arrows in (b). **c**, Four antiferromagnetic domains (*L*_+*X*_, *L*_−*X*_, *L*_+*Y*_, and *L*_−*Y*_ domains). They are distinguished in terms of the orientation of Néel vector **L** (thick magenta arrows) and the corresponding magnetic toroidal moment **T** (thick green arrows). **d** Conceptual illustration of antiferromagnetic trichroism. Absorption coefficient of light propagating along the +*X* axis (orange wave) takes three discrete magnitudes, depending on the direction of **L**, which is conceptually denoted by three colors (blue, red, and white). See text for details.

Here, we report on large visible-light NDD in the ME AFM material Bi_2_CuO_4_ (Fig. 1a, b), which is an insulating version of CuMnAs with four stable Néel vector orientations (Fig. 1c). We demonstrate that the NDD combined with tetragonal symmetry of the crystal structure leads to unconventional magnetically induced trichroism, which we call AFM trichroism (Fig. 1d). Furthermore, using the AFM trichroism, we successfully visualize the field-driven reversal and rotation of the Néel vector.

## Results

### Material and concept of AFM trichroism

Generally, NDD in the (near-)visible range is explained in terms of the interference effect between electric-dipole (E1) and magnetic-dipole (M1) transitions through the spin–orbit interaction^18–20,23,25–27^. This indicates that NDD will be most pronounced when E1 and M1 transitions are comparable in magnitude with each other. However, the E1 transition, which arises due to a lack of inversion symmetry, is usually much stronger than the M1 transition. A clue to solving this problem is obtained from a previous observation^19,23^ of gigantic NDD (more than 100% in a ratio of nonreciprocal-to-reciprocal components in absorption coefficient) in an ME material CuB_2_O_4_. The NDD in CuB_2_O_4_ is dominated by the intra-atomic *d*–*d* crystal field excitations at a Cu ion, which is square-coordinated by four oxygens forming a CuO_4_ plane. Importantly, local inversion symmetry at the Cu site is only slightly broken. This allows a weak E1 transition comparable in magnitude with an M1 transition, thus giving rise to the gigantic NDD^19^. CuB_2_O_4_ is not a fully compensated antiferromagnet but a weak ferromagnet and its NDD appears only in an applied magnetic field. However, as the *d*–*d* crystal-field excitations are largely dominated by a local ligand environment, large spontaneous (zero-field) NDD can be anticipated even in a simple colinear AFM material if it consists of Cu ions with a local ligand environment similar to that in CuB_2_O_4_. To examine this expectation, we explore an AFM material that exhibits linear ME effects and possesses multistable domains, as well as consists of square-planar CuO_4_ units with weak local inversion breaking.

The target material Bi_2_CuO_4_ is an insulating antiferromagnet and has recently attracted attention in terms of possible double Dirac fermions under pressure^28^ and quantum zero-point fluctuation effects on its magnetic anisotropy^29^. The crystal structure (Fig. 1a) belongs to the centrosymmetric tetragonal space group *P*4/*ncc* (ref. ^30^). Hereafter, we refer to the [110], $[\bar{1}10]$ and [001] axes as *X*, *Y*, and *Z*, respectively. Bi_2_CuO_4_ consists of isolated square-planar CuO_4_ units stacked along the *Z* axis in a twisted manner. Significantly, local inversion symmetry at the Cu sites is slightly broken by a small off-center displacement of the Cu ions along the *Z* axis (Fig. 1b). Neutron-scattering experiments^29–31^ have indicated that, below the Néel temperature (*T*_N_ ≈ 44 K), Cu spins form an in-plane colinear spin structure whose direction is parallel to the CuO_4_ plane and more specifically along the *X* (or *Y*) axis^29^. The AFM ordering changes its magnetic point group from 4/*mmm*1ʹ to *mm*ʹ*m*, identical to that in CuMnAs (ref. ^7^). Consequently, the AFM phase may host four equivalent AFM domains specified by the direction of the Néel vector **L** (*L*_+*X*_, *L*_−*X*_, *L*_+*Y*_, and *L*_−*Y*_), as shown in Fig. 1c. Here, the direction of **L** is defined as that parallel to that of Cu1 spins. The linear ME effect was observed in the AFM phase^31^, in agreement with the broken P and T symmetries of the *mm*ʹ*m* group. Therefore, all the above-mentioned requirements for the enhancement of NDD are fulfilled in Bi_2_CuO_4_ with multistable AFM domains.

Furthermore, a combination of the possible NDD and the tetragonal symmetry of the crystal structure can lead to the following characteristic optical phenomenon. As discussed in the literature^7,32^, an AFM phase with the *mmʹm* symmetry possesses a finite magnetic toroidal moment **T** (normal to *mʹ*) with time-reversal-odd polar symmetry^32–34^. Previous studies^15,17–24,26^ have established that a system having a finite **T** exhibits NDD, which is given as a difference in absorption coefficient (*A*) between light beams parallel and antiparallel to fixed **T** or, equivalently, a difference in *A* between +**T** and −**T** states for a fixed light-propagation direction:

1 $${A}_{\rm {NDD}}={A}_{{\bf k}\uparrow \uparrow {\bf T}}-{A}_{{\bf k}\uparrow \downarrow {\bf T}}={A}_{\rm {p}}{\hat{\mathbf k}}\cdot \hat{\mathbf T}$$

where $\hat{{{{{{\bf{T}}}}}}}$ and $\hat{{{{{{\bf{k}}}}}}}$ represent unit vectors parallel to **T** and the light-propagation vector **k**, respectively. The proportionality constant *A*_p_ may depend on angular frequency (*ω*) and polarization (**E**^*ω*^) of light. In Bi_2_CuO_4_, **T** is orthogonal to **L** (see Fig. 1c, Supplementary Fig. 1, and Supplementary Note 1). Consequently, when **k** is parallel to the *XY* plane, Eq. (1) is rewritten as

2 $${A}_{\rm NDD}={A}_{{\bf k}\uparrow \to {\bf L}}-{A}_{{\bf k}\uparrow \leftarrow {\bf L}}={A}_{\rm p}{\hat{\bf e}}_{Z}\cdot (\hat{\bf k}\times \hat{\bf L})$$

where ${\hat{{{{{{\bf{e}}}}}}}}_{Z}$ and $\hat{{{{{{\bf{L}}}}}}}$ denote unit vectors parallel to the +*Z* axis and **L**, respectively. Both **k**↑→**L** and **k**↑${\leftarrow}$**L** represent **k** normal to **L**, but the direction of either **k** or **L** is opposite between **k**↑→**L** and **k**↑${\leftarrow}$**L**. Equation (2) predicts that a single-domain sample of *L*_+*X*_, for example, exhibits three different values of *A*_NDD_ (*A*_p_, −*A*_p_, and 0) when viewed from different principal axes of +*Y*, −*Y*, and +*X/*−*X*, respectively (Supplementary Fig. 2a). Notably, the resulting three-level difference in *A* upon the 90 × *n*° rotation of **k** in the *XY* plane (*n* = 1–3) is purely induced by the AFM order, because this rotation never changes *A* in the paramagnetic phase due to its tetragonal symmetry. Therefore, we term this optical phenomenon AFM trichroism, in analogy with conventional trichroism (or triple absorption) observed in biaxial crystals^35,36^. Furthermore, because the in-plane 90 × *n°* rotation of **k** is equivalent with that of **L** in terms of symmetry, the AFM trichoism can also be viewed as three different absorptions upon the 90 × *n°* rotation of **L** when **k** is fixed along the *X* or *Y* axis (Supplementary Fig. 2b). For example, when *k*_*X*_ > 0 (**k**||+*X*), *A*_NDD_ = *A*_p_ and −*A*_p_ for *L*_+*Y*_ and *L*_−*Y*_, respectively, whereas *A*_NDD_ = 0 for both *L*_+*X*_ and *L*_−*X*_. Thus, the AFM trichroism provides an intriguing possibility that three out of four multi-stable **L** domains can be spatially resolved using the standard optical microscope technique, as conceptually illustrated in Fig. 1d. Based on the linear optics, this imaging method for the multi-stable **L** domains is much simpler and can be faster compared with existing methods such as second-harmonic generation microscopy^10,11^ and X-ray magnetic linear dichroism photoemission electron microscopy^37^.

### NDD in Bi_2_CuO_4_

To demonstrate the possibility of the large NDD and the AFM trichroism, we begin by studying NDD spectra in single crystals of Bi_2_CuO_4_ (see “Methods” and Supplementary Fig. 3 for the sample characterization). For **k**||+*X*, *A*_NDD_ is obtained as a difference in *A* between *L*_+*Y*_ and *L*_−*Y*_ states, because the 180° switching of **L** is equivalent to that of **k**^24,26^, as found from Eq. (2). We therefore prepare single-domain states of *L*_+*Y*_ and *L*_−*Y*_ by cooling the sample from ~50 K (>*T*_N_) while applying magnetic (**H**) and electric (**E**) fields. This so-called ME cooling relies on the linear ME coupling term in the free energy, *α*_*ij*_*H*_*i*_*E*_*j*_. Here, *H*_*i*_ and *E*_*j*_ represent **H** and **E** along the *i* and *j* (*ij* = *X*, *Y*, *Z*) directions, respectively, and *α*_*ij*_ is the linear ME coefficient^13^. In Bi_2_CuO_4_, the *L*_+*Y*_ and *L*_−*Y*_ states have finite *α*_*YZ*_ (and *α*_*ZY*_) with opposite signs^32^ and hence will be stabilized by *H*_*Y*_*E*_*Z*_ > 0 and *H*_*Y*_*E*_*Z*_ < 0, respectively. (For convenience, we assume *α*_*YZ*_ > 0 and *α*_*YZ*_ < 0 for *L*_+*Y*_ and *L*_−*Y*_, respectively). Note that the *L*_+*X*_ and *L*_−*X*_ states have *α*_*YZ*_ = 0 and thus they are not stabilized by *H*_*Y*_*E*_*Z*_ (ref. ^32^).

**Fig. 2:**
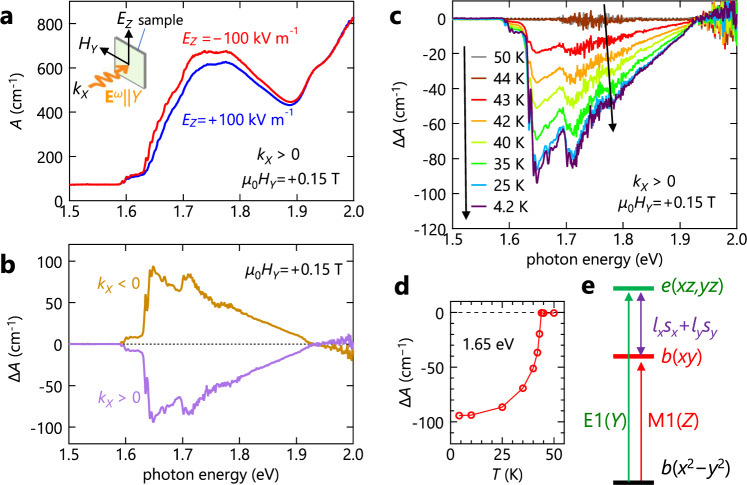
Evidence for nonreciprocal directional dichroism. **a** Absorption coefficient (*A*) spectra (*k*_*X*_ > 0, **E**^*ω*^||*Y*) at 4.2 K. The blue- and the red-colored data were obtained after ME cooling procedure with *E*_*Z*_ = +100 kV m^−1^ and −100 kV m^−1^, respectively, and *μ*_0_*H*_*Y*_ = +0.15 T. The inset shows an experimental geometry. **b** Difference absorption spectra, Δ*A* = *A*(+100 kV/m) − *A*(−100 kV/m), for *k*_*X*_ > 0 (purple) and *k*_*X*_ < 0 (dark yellow). **c** Δ*A* spectra at various temperatures above and below *T*_N_ = 44 K. **d** The temperature dependence of Δ*A* at 1.65 eV, where Δ*A* takes the maximum in the spectra. In (**a**–**d**), the cooling electric and magnetic fields applied during the ME cooling were removed before each measurement. **e** Schematic hole-energy spectrum of Cu(3*d*)-O(2*p*) bonding molecular orbitals of the square-planar CuO_4_^6−^ unit partly reproduced from ref. [^38^]. *a*(*z*^2^) is omitted for clarity. An electric-dipole (magnetic-dipole) transition with a *Y-*(*Z*-)axis electric (magnetic) field of light, allowed in C4 symmetry, is denoted by a green (red) arrow. A spin–orbit coupling term *l*_*x*_*s*_*x*_ + *l*_*y*_*s*_*y*_ hybridizes *b*(*xy*) and *e*(*xz*) [*e*(*yz*)].

Figure 2a shows two absorption spectra of an *X*-plane sample for *k*_*X*_ > 0 at 4.2 K after ME cooling with *μ*_0_*H*_*Y*_ = +0.15 T and *E*_*Z*_ of opposite signs [+100 kV m^−1^ (blue) and −100 kV m^−1^ (red)]. The light polarization is set along the *Y* axis (**E**^*ω*^||*Y*). To observe spontaneous effects, the cooling magnetic and electric fields were removed before each measurement. It is seen that both spectra start to increase above 1.6 eV, form a broad band centered at approximately 1.75 eV, and then become more intense above 1.9 eV. At temperatures below *T*_N_, fine structures develop at photon energies below ~1.7 eV in both spectra (see the inset in Supplementary Fig. 4), although their physical origins are not yet established. The most significant observation here is that the two spectra exhibit a marked difference between 1.6 and 1.9 eV, which is more evident in the difference spectrum, Δ*A* = *A*(+100 kV m^−1^) − *A*(−100 kV m^−1^), as displayed in Fig. 2b (purple curve). This finite Δ*A* is the first evidence for the presence of the NDD. Moreover, the **k** and **L** odd nature of *A*_NDD_ expected from Eq. (2) is ensured by the fact that the Δ*A* spectrum is completely reversed upon a reversal of either *k*_*X*_ (Fig. 2b) or *L*_*Y*_ with a negative cooling *H*_*Y*_ (Supplementary Fig. 5). Furthermore, Δ*A* emerges only in the AFM phase (below *T*_N_) with a finite **L** (Fig. 2c, d). These results demonstrate that Δ*A* arises from NDD, that is, Δ*A* = *A*_NDD_ [Eq. (2)].

The magnitude of the observed Δ*A* is very large. In particular, the magnitude at 1.65 eV reaches a value as large as 90 cm^−^^1^ and a relative difference Δ*A*/*A*_ave_ exceeding 40%, where *A*_ave_ is the average of *A*(+100 kV m^−1^) and *A*(−100 kV m^−1^) (Supplementary Fig. 6). This relative difference is the largest among the reported OME effects in the (near-)visible light region for antiferromagnets [cf. ~0.02% in Cr_2_O_3_ (ref. ^16^) and ~4% in Pb(TiO)Cu_4_(PO_4_)_4_ (ref. ^27^)]. Therefore, we have experimentally demonstrated that the material exploration based on the weak inversion asymmetric CuO_4_ square-planar unit is an efficient way to achieve large visible-light NDD in the in-plane colinear AFM material.

### Possible origin of the NDD

To discuss the possible microscopic origin of the large NDD in Bi_2_CuO_4_, it is convenient to begin with electronic energy levels of the square-planar CuO_4_^6−^ unit proposed in ref. ^38^ (see Supplementary Fig. 7a). The square-planar crystal field splits five 3*d* orbitals of the Cu^2+^ ion into ${d}_{{z}^{2}}$, ${d}_{{x}^{2}-{y}^{2}}$, ${d}_{xy}$ and ${d}_{xz}({d}_{yz})$. Here, *x*, *y*, and *z* represent local coordinate axes at the Cu site, with *z* being parallel to the crystallographic *Z* axis. Due to the strong covalency of the Cu(3*d*)-O(2*p*) bond in Bi_2_CuO_4_, each 3*d* orbital is hybridized with particular 2*p* orbitals of the four surrounding oxygens, forming bonding and antibonding molecular orbitals. On the basis of the C4 symmetry at the Cu site, we label bonding orbitals as *b*(*x*^2^–*y*^2^), *b*(*xy*), *a*(*z*^2^) and *e*(*xz*) [*e*(*yz*)], which belong to the irreducible representations of B, B, A, and E, respectively. Bonding-orbital levels in a hole picture, mainly reproduced from ref. ^38^, are depicted in Fig. 2e. [*a*(*z*^2^) is omitted for clarity since it is both E1- and M1-forbidden (see Supplementary Fig. 7)]. The ground state is *b*(*x*^2^–*y*^2^) (formed by ${d}_{{x}^{2}-{y}^{2}}$), whereas the other four excited levels are located in the energy region from 1.4 to 1.7 eV, which roughly coincides with the region where the broad absorption bands are observed (Fig. 2a). Thus, the observed absorption modes in 1.6–1.9 eV are attributable to the intra-cluster transitions between the 3*d*–2*p* hybridized orbitals. The increasing absorption above 1.9 eV is likely ascribed to a charge-transfer excitation to bonding orbitals formed by oxygen 2*p* orbitals (see Supplementary Fig. 7a). Group theory indicates that a transition from *b*(*x*^2^−*y*^2^) to *b*(*xy*) is E1-allowed with **E**^*ω*^||*Z*, while that to *e*(*xz*) and *e*(*yz*) is E1-allowed with **E**^*ω*^||*Y*. Our measurements show that the absorption for **E**^*ω*^||*Z* is stronger than that for **E**^*ω*^||*Y* at around 1.6–1.7 eV, while conversely weaker at around 1.7–1.9 eV (Supplementary Fig. 8). This suggests that the *b*(*xy*) state and the *e*(*xz*) and *e*(*yz*) states are located at around 1.6–1.7 eV and 1.7–1.9 eV, respectively. In the following, we pay our attention to the energy region of 1.6–1.7 eV, where we observe the largest NDD.

As mentioned above, the NDD in the (near-)visible range is explained in terms of the interference between E1 and M1 transitions, which is expressed as^19,23,25,26^

3 $${\alpha }_{{{{{\rm{NDD}}}}}} 	={\alpha }_{+k}-{\alpha }_{-k}\propto {|\langle e|{H}_{\rm E1}+{H}_{\rm M1}|g\rangle |}^{2}-{|\langle e|{H}_{\rm E1}-{H}_{\rm M1}|g\rangle |}^{2}\\ 	=4{{{{\mathrm{Re}}}}}[\langle {\it{g}}|{\it{H}}_{\rm E1}|{\it{e}}\rangle \langle {\it{e}}|{\it{H}}_{\rm M1}|{\it{g}}\rangle ]$$

where *g* (*e*) represents the wave function of the ground (excited) state and *H*_E1_ (*H*_M1_) denotes the electric-dipole (magnetic-dipole) transition operator. This indicates that NDD appears only when the transition *g* → *e* is both E1- and M1-allowed. Also, Eq. (3) explicitly indicates that a spin–orbit interaction (SOI), *H*_SO_ = *λ*(**l**·**s**) = *λ*(*l*_*x*_*s*_*x*_ + *l*_*y*_*s*_*y*_ + *l*_*z*_*s*_*z*_), plays a critical role for NDD^25^ because $\langle g|{H}_{\rm E1}|e\rangle$ and $\langle e|{H}_{\rm M1}|g\rangle$ in the absence of SOI are purely real and imaginary, respectively, yielding ${{{{\mathrm{Re}}}}}[\langle {g}|{H}_{\rm {E1}}|{e}\rangle \langle {e}|{H}_{\rm {M1}}|{g}\rangle ]=0$. Here, **l** and **s** are the orbital and spin angular operators, respectively, and *λ* is the spin-orbit coupling constant. In our experiments with **E**^*ω*^||*Y* and an oscillating magnetic field of light parallel to the *Z* axis (**H**^*ω*^||*Z*), the transition *b*(*x*^2^−*y*^2^) → *b*(*xy*) is M1-allowed but E1-forbidden in the absence of the SOI. When the SOI is switched on, the E1 allowed *e*(*xz*) [*e*(*yz*)] state is mixed into *b*(*xy*) via the *λ*(*l*_*x*_*s*_*x*_ + *l*_*y*_*s*_*y*_) term with E symmetry. (The *λl*_*z*_*s*_*z*_ term with A symmetry does not allow such a hybridization.) As a result, the transition from *b*(*x*^2^−*y*^2^) to the modulated *b*(*xy*) state becomes both E1 and M1 allowed through the SOI (Fig. 2e and Supplementary Fig. 7c); hence, NDD can emerge.

The fairly large NDD observed in Bi_2_CuO_4_ can be ascribed to, first of all, the weak inversion symmetry breaking at the Cu site (Fig. 1b). This can make the E1 transition small and comparable in magnitude with the M1 transition, which enhances the E1-M1 interference effect. Essentially the same scenario has been proposed for the above-mentioned gigantic NDD in CuB_2_O_4_ (ref. ^19^) (see Supplementary Fig. 9 and Supplementary Note 2 for comparisons of NDD between Bi_2_CuO_4_ and CuB_2_O_4_ in terms of the magnitude and a light-polarization dependence). In addition, because the SOI energy scale set by *λ* ~ 0.1 eV for the Cu ion is comparable to the energy difference (0.2 ~ 0.3 eV) between the *b*(*xy*) and the *e*(*xz*) [*e*(*yz*)] state, the hybridization between these states can be significant, which may also contribute to the enhancement of the NDD. Finally, because the spin state of the excited states [*b*(*xy*), *e*(*xz*), and *e*(*yz*)] is identical to that of the ground state (since the E1 and M1 transitions considered here preserve the spin state), the orbital hybridization via the *l*_*x*_*s*_*x*_ + *l*_*y*_*s*_*y*_ term contributes to the optical process only when the ground state has the *x*-(*y*-)axis spin component that is preserved upon the operation of *l*_*x*_*s*_*x*_ (*l*_*y*_*s*_*y*_). Thus, the in-plane (*xy*-plane) nature of the Cu spins in Bi_2_CuO_4_ also contributes to the enhancement of the NDD.

### Imaging of the Néel vector and its electric-field reversal

**Fig. 3:**
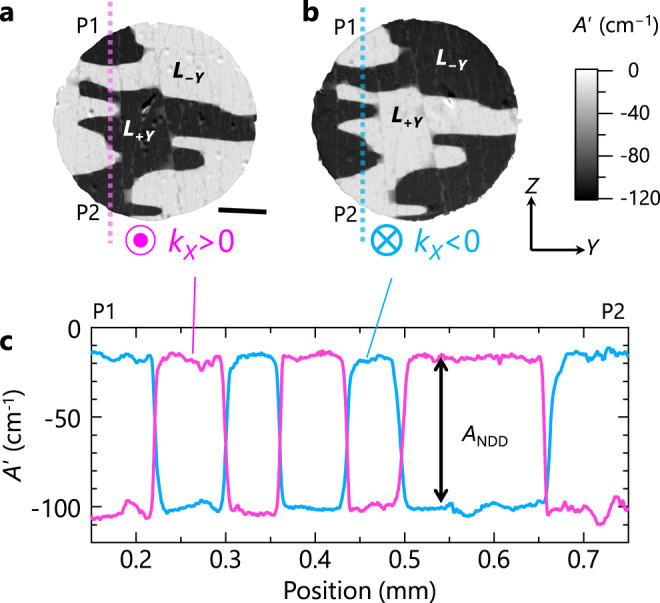
Antiferromagnetic domains after zero-field cooling. **a**, **b** Two-dimensional spatial distribution of *A*ʹ for *k*_*X*_ > 0 (**a**) and *k*_*X*_ < 0 (**b**) of an *X*-plane sample at 5 K and at 0 T after zero-field cooling. Here, *A*ʹ is the variation of absorption coefficient from 50 K (> *T*_N_). **E**^*ω*^ is parallel to the *Y* axis and light wavelength is 750 nm. Dark and bright regions in (a) correspond to the *L*_+*Y*_ and *L*_−*Y*_ domains, respectively, while those in (b) correspond to the *L*_−*Y*_ and *L*_+*Y*_ domains, respectively. Scale bar below (**a**) is 0.2 mm. **c** Profiles taken along the same line from P1 to P2 in (**a**) (magenta) and (**b**) (sky blue), respectively. The difference between the two profiles at the same position corresponds to nonreciprocal directional dichroism, *A*_NDD_.

Taking advantage of the large NDD, we visualize the spatial distribution of **L** in a crystal with optical microscopy. In the experiments, the spatial distribution of the transmitted light intensity *I *from an *X*-plane sample (i.e., **k**||*X*) is recorded using a CMOS camera. Subtracting *I* at 50 K (*I*_50K_) as a paramagnetic reference, we can obtain an effective absorption coefficient *A*ʹ (i.e., the variation of *A* from 50 K), $A^{\prime} =A-{A}_{\rm 50K}=-[{{{{\mathrm{ln}}}}}({\it{I}}/{\it{I}}_{\rm 50K})]/{\it{d}}$, where *d* is the sample thickness. Note that the difference between *A*ʹ for *k*_*X*_*L*_*Y*_ > 0 and *k*_*X*_*L*_*Y*_ < 0 corresponds to *A*_NDD_. To obtain the maximum *A*_NDD_, we choose **E**^*ω*^||*Y* and a wavelength of 750 nm (which corresponds to a photon energy of 1.65 eV) (see Fig. 2b–d). Figure 3a displays an *A*ʹ image of an *X*-plane sample for *k*_*X*_ > 0 at 5 K and 0 T after zero-field cooling, showing the strong contrast between the two discrete levels. Moreover, the contrast is reversed for *k*_*X*_ < 0 (Fig. 3b) and, as seen in the profiles of *A*ʹ taken along the same line (from P1 to P2) shown in Fig. 3c, the reversed component in most of the positions amounts to ~80 cm^−1^, comparable with *A*_NDD_ ~90 cm^−1^ at 1.65 eV (Fig. 2b). This means that the dark and bright regions for *k*_*X*_ > 0 correspond to the *L*_+*Y*_ and *L*_−*Y*_ domains, respectively, and they are distributed uniformly along the depth direction (otherwise the contrast would be weaker). The same domain pattern, albeit with a weaker contrast, is observed for **E**^*ω*^||*Z* and unpolarized light (Supplementary Fig. 10). Unexpectedly, the *L*_+*X*_ and *L*_−*X*_ domains seem to be absent. The value of *A*ʹ (**E**^*ω*^||*Y*) for these domains is expected to be −60 cm^−1^, which is the middle of −20 and −100 cm^−1^ for the *L*_+*Y*_ and *L*_−*Y*_ domains (Fig. 3c). However, no region with such an *A*ʹ value is found in the images.

**Fig. 4:**
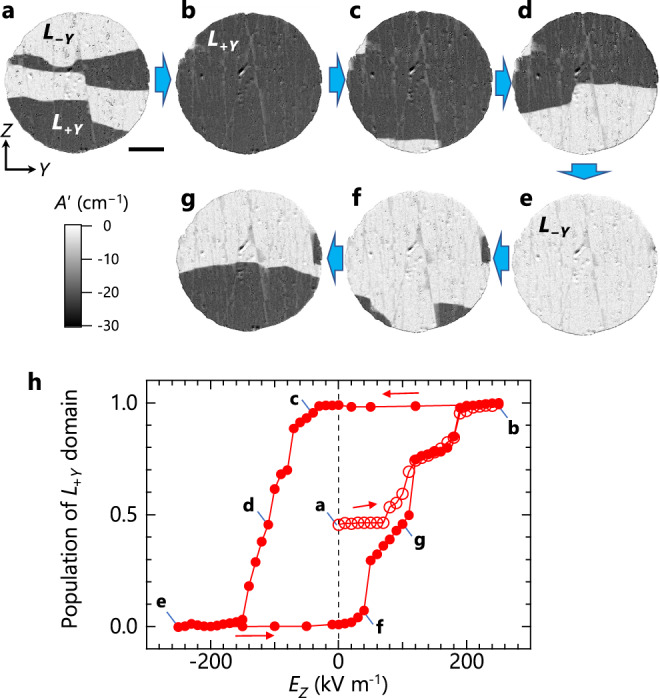
Visualization of the Néel vector reversal with an electric field. **a–g** Selected *A*ʹ images of an *X*-plane sample for *k*_*X*_ > 0 at 43.7 K (< *T*_N_) in a cyclic electric field (*E*_*Z*_ = 0 → +250 → −250 → +250 kV m^−1^) with a constant bias magnetic field (*μ*_0_*H*_*Y*_ = 0.15 T). Here, *A*ʹ is the variation of absorption coefficient from 50 K (> *T*_N_). Dark and bright regions correspond to *L*_+*Y*_ and *L*_−*Y*_ domains, respectively. Scale bar below (**a**) is 0.2 mm. **h** The *E*_*Z*_ dependence of population of *L*_+*Y*_ domains derived from the areal ratio of dark and bright regions in a series of *A*ʹ images. The data points with alphabets (**a**–**g**) correspond to the images (**a**)–(**g**), respectively.

After identifying the domain states, we examine the electric-field switching of **L** through the ME coupling. First, we cool the sample to 43.7 K (<*T*_N_) at zero field and subsequently apply a small bias field (*μ*_0_*H*_*Y*_ = 0.15 T) to obtain a finite switching force (*H*_*Y*_*E*_*Z*_). At this initial state, both the *L*_+*Y*_ (dark) and *L*_−*Y*_ (bright) domains are present (Fig. 4a). Then, we investigate the evolution of the domains under the application of an electric field (*E*_*Z*_ = 0 → +250 → −250 → +250 kV m^−1^). Selected images and the extracted population of the *L*_+*Y*_ domain are shown in Fig. 4b–g and h, respectively. Figure 4h clearly demonstrates that the application of *E*_*Z*_ leads to single-domain states [*L*_+*Y*_ (Fig. 4b) and *L*_*−Y*_ (Fig. 4e)], evidencing a complete *E*_*Z*_-driven **L** reversal. It is also observed that nucleation of opposite domains occurs only at the sample edges (Fig. 4c, f), and only the *L*_+*Y*_ and *L*_−*Y*_ domains appear throughout the reversal (Fig. 4d, g). Thus, the **L** reversal in Bi_2_CuO_4_ is dominated by 180° AFM domain-wall (AFDW) motion.

### Demonstration of AFM trichroism

**Fig. 5:**
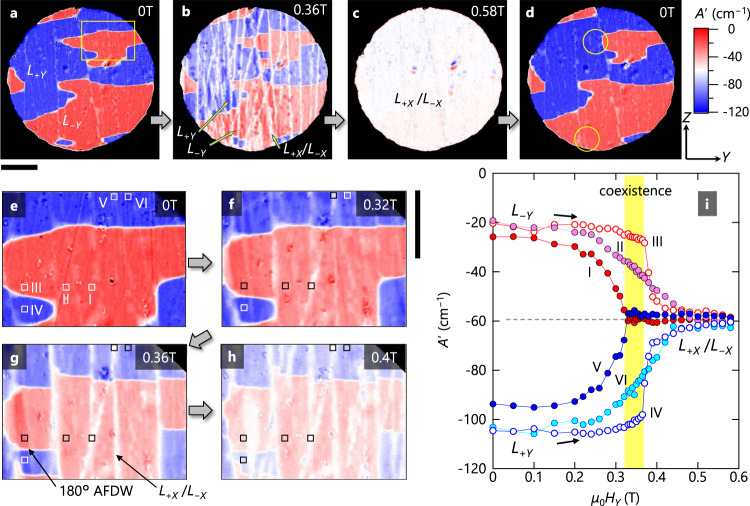
Demonstration of antiferromagnetic trichroism. **a**–**d** A sequence of *A*ʹ images of an *X*-plane sample for *k*_*X*_ > 0 at 5 K in varying applied fields (*μ*_0_*H*_*Y*_) of 0 T (**a**), 0.36 T (**b**), 0.58 T (**c**), and then 0 T (**d**). Here, *A*ʹ is the variation of absorption coefficient from 50 K (> *T*_N_). **E**^*ω*^ is parallel to the *Y* axis and light wavelength is 750 nm. The domain pattern at 0 T is slightly changed once *μ*_0_*H*_*Y*_ of 0.58 T is applied, as denoted by yellow circles in (d). Scale bar below (a) is 0.2 mm. **e**–**h** A sequence of magnified images of the yellow-box region in (a) in varying *μ*_0_*H*_*Y*_ of 0 T (**e**), 0.32 T (**f**), 0.36 T (**g**), and 0.4 T (**h**). In (**g**), a boundary separating *L*_+*Y*_ (blue) and *L*_−*Y*_ (red) domains corresponds to a 180° antiferromagnetic domain wall (AFDW). A *L*_+*X*_/*L*_−*X*_ domain (*L*_+*X*_ or *L*_−*X*_ or both) is pointed by a black arrow. Scale bar next to (**f**) is 0.1 mm. **i** The *μ*_0_*H*_*Y*_ dependence of *A*ʹ at regions I–VI defined in (**e**). The yellow rectangle represents a region where the *L*_+*Y*_, *L*_−*Y*_, and *L*_+*X*_/*L*_−*X*_ domain states coexist.

A promising strategy to create the *L*_+*X*_ and *L*_−*X*_ domains, which is vital for the demonstration of the AFM trichroism, is an application of *H*_*Y*_, because **L** tends to orient perpendicular to the external **H**. Indeed, a related anomaly is observed in a magnetization curve at ~0.2 T (Supplementary Fig. 3). Figure 5a–c shows a series of *A*ʹ images in applied fields of 0, 0.36, and 0.58 T. A complete set of *A*ʹ images and a real-time video of raw images are provided in Supplementary Movies 1 and 2, respectively. At 0.58 T, the image is almost uniform. The *A*ʹ value approximately coincides with the expected value (−60 cm^−1^) for the *L*_+*X*_/*L*_−*X*_ domains (i.e., *L*_+*X*_ or *L*_−*X*_ or both), indicating the existence of the *L*_+*X*_/*L*_−*X*_ domains. This is further evidenced by a separate imaging experiment with slightly oblique light (Supplementary Fig. 11 and Supplementary Note 3). Therefore, we experimentally demonstrate three different absorptions upon the in-plane 90 × *n*° rotation of **L**, that is, the AFM trichroism.

Furthermore, the AFM trichroism reveals a characteristic field evolution of the **L** domains. Figure 5e–h shows magnified images of the region surrounded by a yellow box in Fig. 5a at selected *H*_*Y*_. In Fig. 5i, we also plot the *μ*_0_*H*_*Y*_ dependence of *A*ʹ in the representative regions labeled as I–VI (Fig. 5e). It is seen that *A*ʹ in the majority regions (II and VI) gradually changes above ~0.26 T, and the transformation to the *L*_+*X*_/*L*_−*X*_ domains is complete at ~0.45 T. By contrast, the domain transformation in stripe-shaped regions (I and V) is complete at a smaller field of 0.3 T, whereas the *L*_+*Y*_ and *L*_−*Y*_ domains in the respective regions III and IV survive up to about 0.36 T. Thus, the *L*_+*X*_/*L*_−*X*_ domains evolve in a highly inhomogeneous manner, yielding the coexistence of *L*_+*Y*_, *L*_−*Y*_, and *L*_+*X*_/*L*_−*X*_ domains between 0.31 and 0.36 T.

Finally, we discuss several unusual features of the domain states obtained through the AFM trichroism. The first feature is the absence of the *L*_+*X*_/*L*_−*X*_ domains at 0 T, which contradicts the energy equivalence of the four domains. In the imaging experiments, the *X* plane of the crystal is fixed to a copper substrate. Likely, a difference in thermal expansion between the sample and the substrate causes a stress on the sample only within the *X* plane, and the resulting anisotropic thermal stress breaks the original domain degeneracy. The thermal stress could also explain the discrepancy in the domain-transformation field observed in magnetization (~0.2 T) and imaging experiments (~0.35 T), since it should depend on the way the sample is mounted (see Methods). The second feature is the inhomogeneous field-induced domain transformation; it starts to grow from stripe-shaped regions with a slightly weaker contrast at 0 T (I and V) than other regions (Fig. 5e–h). We speculate that these regions contain a small fraction of the *L*_+*X*_/*L*_−*X*_ domains along the depth direction, which act as a seed for the full growth of the *L*_+*X*_/*L*_−*X*_ domains. The weaker contrast regions repeatedly appear at approximately the same positions after heating the sample to room temperature (compare Figs. 3a and 5a) and cannot be eliminated by ME cooling (Supplementary Fig. 12). Thus, they likely originate from internal defects. The third feature is the memory effect: the domain pattern at 0 T remains unchanged after applying *μ*_0_*H*_*Y*_ = 0.58 T (compare Fig. 5a and d), except for the yellow circular regions. This suggests that the domain transformation is dominated by a Néel vector rotation within each domain (see also Supplementary Fig. 11 and Supplementary Note 3). The last feature is the field-induced change in the shape of the boundary separating the *L*_+*Y*_ and *L*_−*Y*_ domains, i.e., 180° AFDW (Fig. 5e–g). The 180° AFDWs are curved at 0 T, but they become straight perpendicular to the *Z* axis upon applying *H*. Such a field-induced change of the 180° AFDW is unusual, and whether it is intrinsic to antiferromagnets with multistable domain states is an interesting future subject.

In conclusion, through the strategic material exploration based mainly on the square coordinated Cu sites with the weak local inversion breaking, we have successfully achieved the large spontaneous nonreciprocal directional dichroism exceeding 40% at 1.65 eV in the insulating antiferromagnetic material Bi_2_CuO_4_, which possesses the four equivalent Néel vector orientations. Moreover, we have demonstrated that the combination of the nonreciprocal directional dichroism and the tetragonal symmetry of the crystal structure in Bi_2_CuO_4_ leads to the unconventional antiferromagnetically induced trichroism. Furthermore, we have shown that this antiferromagnetic trichroism enables the visualization of the field-driven reversal and reorientation of the Néel vector. We stress that the concept of antiferromagnetic trichroism is general and extendable to a broad class of magnetoelectric antiferromagnets with high crystal symmetry (trigonal, tetragonal, hexagonal, and cubic). The present work will stimulate further efforts to explore large nonreciprocal optical functionalities in antiferromagnets and understand the complex physics underlying multistable antiferromagnetic domains, which may contribute to the design of electric-field-controllable and optically readable higher-density memories.

## Methods

### Sample preparation and characterization

Single crystals of Bi_2_CuO_4_ were grown using a laser-based floating-zone furnace composed of a five laser-head design (Quantum Design LFZ1A)^39^. A typical growth rate was 2.0 mm h^−1^ and a counter-rotation speed was 10 rpm both for feed and seed rods. Crystal growth was performed under flowing pure oxygen. At the initial several hours of the crystal growth, a laser current was tuned manually in the range between 26.9 and 27.4 A in order to stabilize a molten zone. Subsequently, an automatic constant-temperature mode was utilized, which allows for a highly stable crystal growth for more than 24 hours without any manual tuning. The obtained single-crystalline rods were very easily cleaved parallel to the *Z* plane. The cleavage plane is very shiny (see the inset of Supplementary Fig. 3), indicating high crystallinity. No impurity phase was detected in powder X-ray diffraction patterns of crushed crystals. The orientation in the *Z* plane was determined by the Laue X-ray method. Magnetization (*M*) as functions of temperature (*T*) and magnetic field (*H*) was measured on a plate-like sample whose widest face is parallel to the *Z* axis by using a commercial SQUID magnetometer (MPMS, Quantum Design). The *T* dependence of *M* confirms that the antiferromagnetic transition takes place at *T*_N_ = 44 K (Supplementary Fig. 3), in agreement with previous studies^30,31^. The crystal structure displayed in Fig. 1a was drawn by using VESTA software^40^.

### Optical absorption measurements

Optical absorption spectra in the photon energy range of 1.2 < *E*_ph_ < 3.1 eV were measured using a homebuilt fiber-based optical system whose design is similar to that reported previously^41^. Our system can be inserted into a commercial physical property measurement system (PPMS, Quantum Design), which allows for a control of sample temperature and an application of a magnetic field. Light from a tungsten–halogen lamp (AvaLight-HAL-S-MINI, Avantes) was guided using an optical fiber to a sample, and then the light transmitted through the sample was guided using a different optical fiber to a spectrometer (Flame-S, Ocean Insight) with an optical resolution of 1.5 nm. A plate-like sample with the widest face parallel to the (110) plane (*X* plane) was used for the optical absorption measurements. A pair of the sample surfaces was polished with lapping films. A thickness (*d*) of the sample was about 100 μm. With this thickness, the absorption spectra at *E*_ph_ > 2.0 eV cannot be measured due to large absorption. Reducing the sample thickness was unsuccessful due to the above-mentioned cleavage nature. To apply an electric field, a pair of parallel electrodes with a 1 mm gap was made on one side of the sample surfaces using conductive silver paste. The electric field was generated by a voltage source (Keithley 6517). Light polarization was controlled by a wire-grid polarization film (Asahi Kasei WGFTM), which was placed on the optical path in front of the sample. When changing the direction of light polarization and/or switching the light-propagation direction, we took out the optical system from the PPMS cryostat, reoriented the polarization film, and then reinstalled the system.

### Optical domain imaging

Domain-imaging experiments were performed using a homebuilt horizontal polarized microscope in the transmittance geometry^27^. As an illumination source, we used a monochromatic LED (M730L5, Thorlabs) combined with a band-pass filter of 750 nm (FWHM = 10 nm). Microscopic images were taken by a scientific CMOS camera (Quantalux sCMOS camera, Thorlabs) with an exposure time of 100 milliseconds or less. The spatial resolution of the microscope is better than 4 μm (ref. ^27^). A plate-like sample (*d* ≈ 100 μm) whose widest face is parallel to the *X* plane was used. The sample was glued using a silver paste on an oxygen-free copper plate with a hole for light transmission, which was then mounted on the cold head of a helium-flow cryostat (MicrostatHe, Oxford Instruments). A magnetic field was generated by an electromagnet (3480, GMW Associates). To apply an electric field to the *X*-plane sample, a voltage source (Keithley 6517) was connected to a pair of parallel electrodes formed on a front surface of the sample. The gap distance of the pair electrodes was about 1 mm.

## Supplementary information

Supplementary Information

Description of Additional Supplementary Files

Supplementary Movie 1

Supplementary Movie 2

## Data Availability

The source data for one-dimensional plots shown in Figs. 2–5 and Supplementary Figs. 3–6,8,9 are provided as a Source Data file. The source numerical data for optical microscopy images are available from the corresponding author upon reasonable request. Source data are provided with this paper.

## Source data

Source Data
