# Supplementary material for: Visualizing rotation and reversal of the Néel vector through antiferromagnetic trichroism
Source: Nat Commun. 2022 Feb 4;13:697. doi: 10.1038/s41467-022-28215-w (PMC8816959; doi:10.1038/s41467-022-28215-w)
Supplement: Supplementary file 1 — Supplementary Information [file 41467_2022_28215_MOESM1_ESM.pdf]

Supplementary Information for

"Visualizing rotation and reversal of the Néel vector through antiferromagnetic trichroism"

Kenta Kimura,[1]* Yutaro Otake,[2] and Tsuyoshi Kimura[1]

[1]*Department of Advanced Materials Science, University of Tokyo, Kashiwa 277-8561, Japan*
[2]*Department of Applied Physics, University of Tokyo, Hongo, 7-3-1, Bunkyo, Tokyo 113-8656, Japan*

*To whom correspondence should be addressed. E-mail: kentakimura@edu.k.u-tokyo.ac.jp

Supplementary Figures

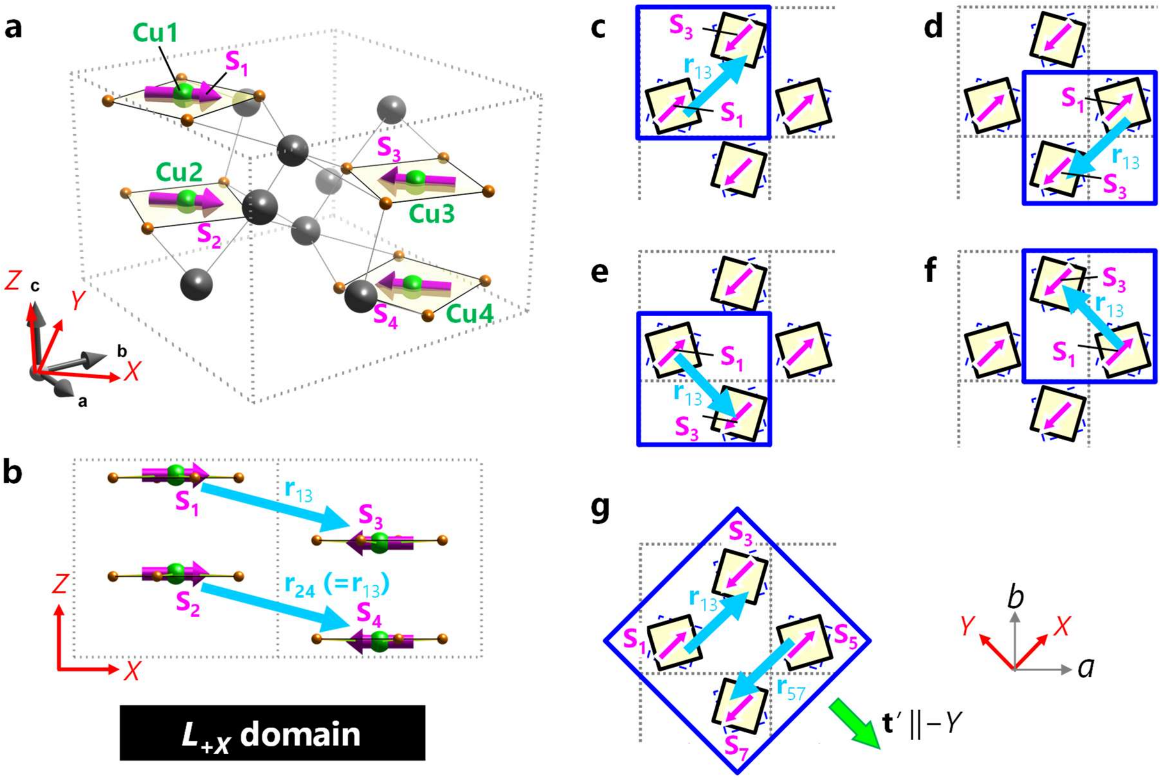

Supplementary Figure 1: Calculation of microscopic magnetic toroidal moment of the L_{+X} domain in Bi_2CuO_4. a,**b**, Three-dimensional (**a**) and *Y*-axis (**b**) views of crystal and magnetic structures with the labels of Cu ions and spins. The gray dotted lines denote a primitive unit cell. The sky-blue arrows represent position vectors from Cu1 to Cu3, $\mathbf{r}_{13} \equiv \mathbf{r}_1 - \mathbf{r}_3$, and from Cu2 to Cu4, $\mathbf{r}_{24} \equiv \mathbf{r}_2 - \mathbf{r}_4$ (= $\mathbf{r}_{13}$). **c**-**f**, Top-views of magnetic structures which differ in the choice of the primitive unit cell (blue squares). **g**, Top-view of magnetic structure with a unit cell (blue square) doubled compared to the primitive unit cell. The green arrow denotes the direction of the microscopic magnetic toroidal moment, $\mathbf{t}'$, obtained from the calculation using the unit cell.

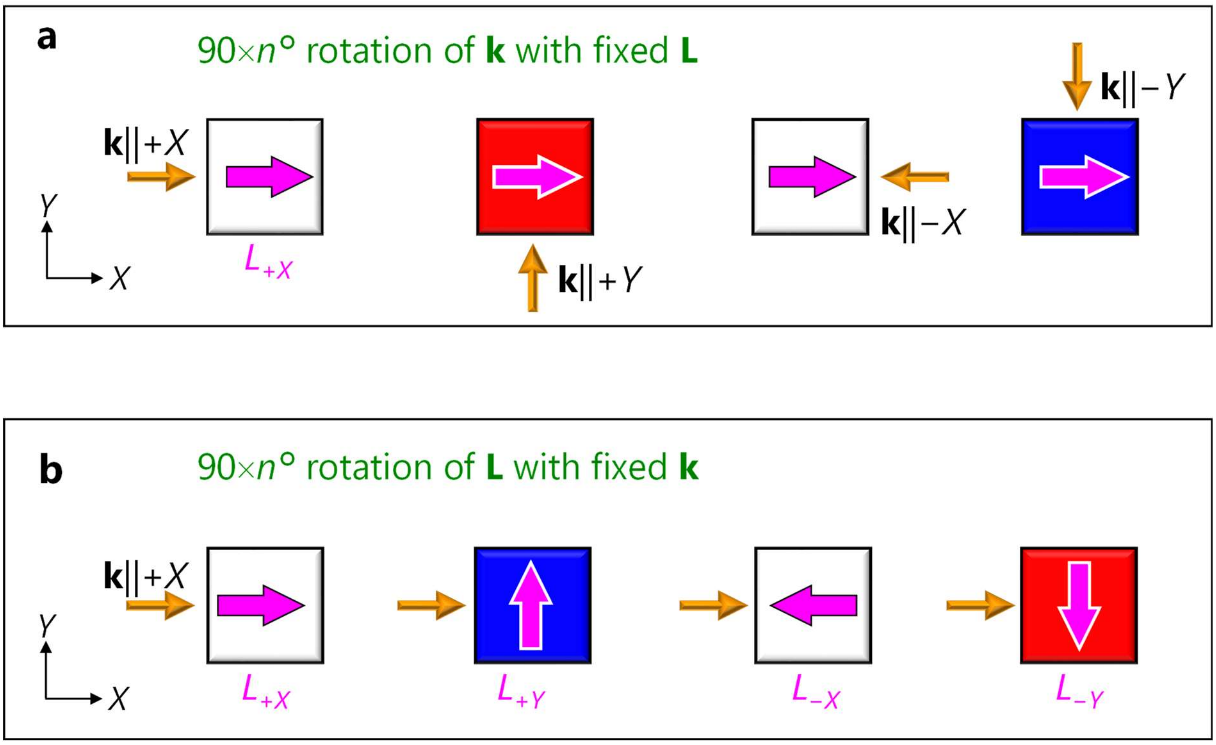

Supplementary Figure 2: Antiferromagnetic trichroism in Bi_2CuO_4. (a) Due to the nonreciprocal directional dichroism (NDD) in the antiferromagnetic (AFM) phase, a single AFM domain sample exhibits three different values of absorption coefficient A when viewed from three different principal axes, which we term AFM trichroism. For the single domain state with the Néel vector L_{+X} (denoted by thick magenta arrows), for example, contributions of the NDD to A (A_{NDD}) are $+A_p$, $-A_p$ and 0, respectively, when viewed from the $+Y$, $-Y$ and $+X/-X$ axes. The three different values of A_{NDD} are represented by red ($+A_p$), blue ($-A_p$) and white (0) colors. Here, A_p is the proportional constant defined in Eq. (1) of the main text. Note that the AFM trichrosim in (a) is viewed as the three different values of A upon the $90 \times n^{\circ}$ rotation of the light propagation vector **k** in the XY plane (n = 1-3). (**b**) Because this in-plane $90 \times n^{\circ}$ rotation of **k** is equivalent to that of **L** in terms of symmetry, the AFM trichoism can also be viewed as three different absorptions upon the $90 \times n^{\circ}$ rotation of **L** (a switching of AFM domains) when **k** is fixed along the X or Y axis. For example, when $k_X > 0$ (**k**||+X), $A_{NDD} = +A_p$ (blue) for L_{+Y}, $A_{NDD} = -A_p$ (red) for L_{-Y} and $A_{NDD} = 0$ for both L_{+X} and L_{-X}.

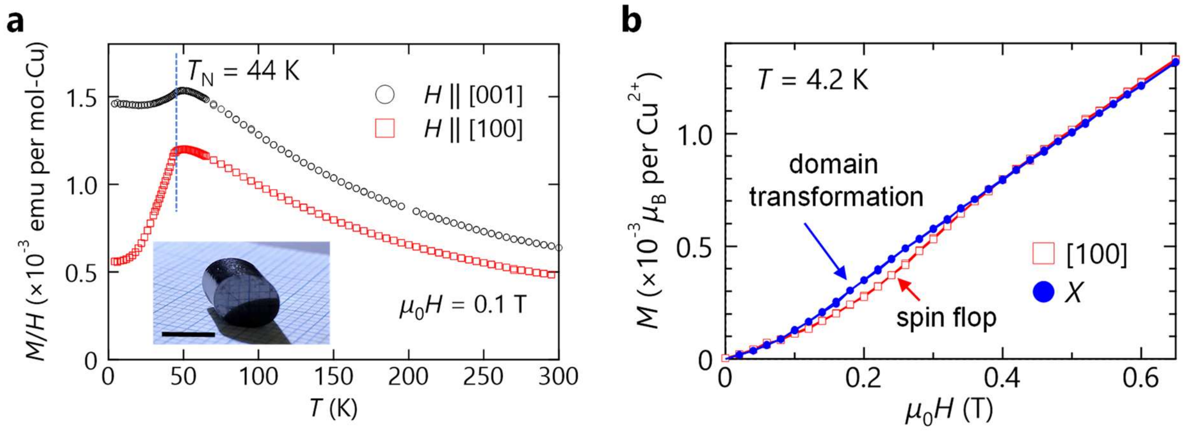

Supplementary Figure 3: Magnetic properties of a Bi_2CuO_4 single crystal. **a**, The temperature (T) dependence of magnetization (M) divided by a magnetic field (H) applied along the [001] (circle) and the [100] (square) axes. A kink at $T_N = 44$ K seen in both the axes indicates the antiferromagnetic transition. A picture of a grown crystal is displayed in the inset (scale bar, 5 mm). **b**, Magnetization curves at 4.2 K for H along the [100] (red) and the X (blue) directions. The magnetization curves for $H_{[100]}$ shows a nonlinear increase at around 0.3 T. This magnetization anomaly is attributed to a spin-flop transition in which spins in all four domains are reoriented toward the direction perpendicular to H (Supplementary Refs. 1 and 2). A nonlinear increase is also seen in the magnetization curves for H_X at around 0.2 T. As discussed in the main text, this is attributable to a domain transformation from domains with spins parallel to H to those perpendicular to H. The same crystal was used for all the measurements in (a) and (b).

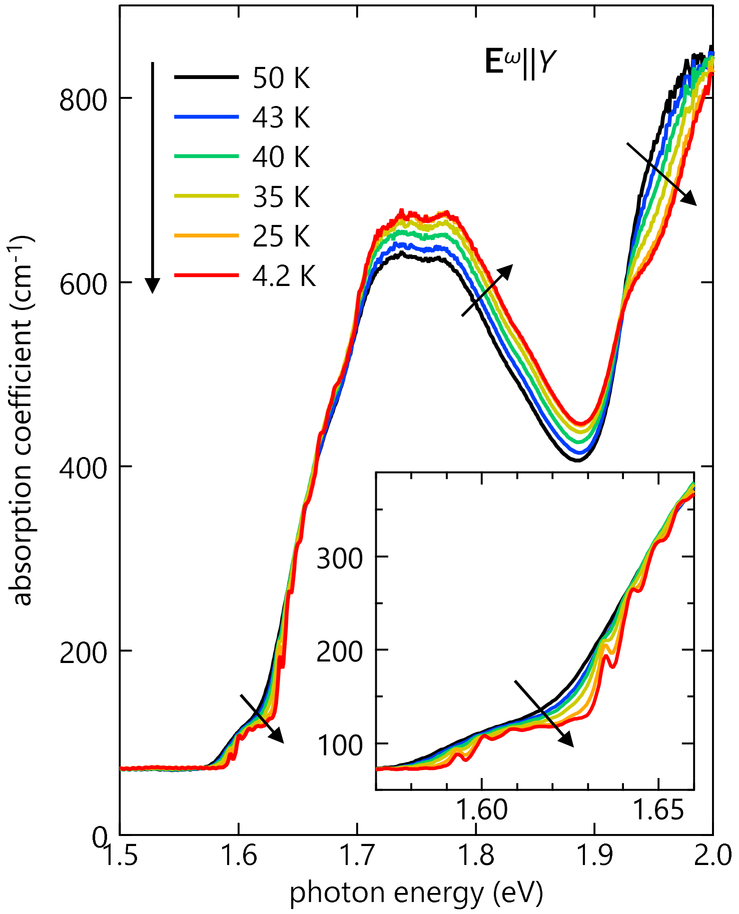

Supplementary Figure 4: Absorption spectra at various temperatures above and below $\boldsymbol{T_N}$**.** Light propagation vector **k** is parallel to the $+X$ axis ($k_X > 0$) and linear polarization of the light is parallel to the Y axis ($\mathbf{E}^{\omega}||Y$). The inset shows a magnified view between 1.57 and 1.66 eV. Fine structures in the spectra develop below T_N.

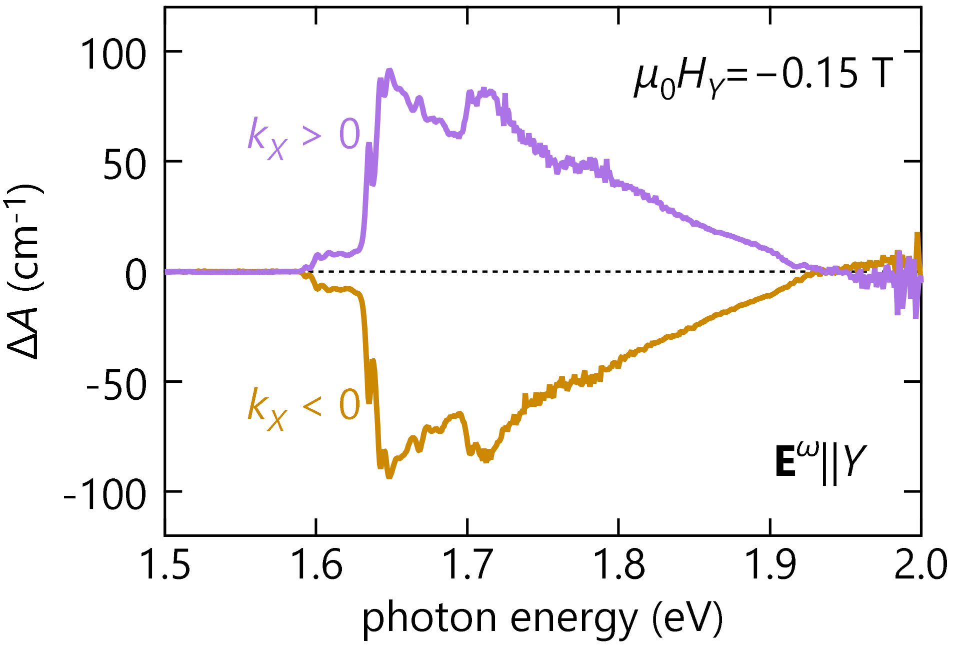

Supplementary Figure 5: Nonreciprocal directional dichroism for a negative cooling magnetic field. The spectra of ΔA [$= A(+100\ \mathrm{kV\ m^{-1}}) - A(-100\ \mathrm{kV\ m^{-1}})$] for $k_X > 0$ (purple) and $k_X < 0$ (dark yellow) were obtained by the same procedure as those in Fig. 2b of the main text, except for the sign of a cooling magnetic field. Here, $\mu_0 H_Y = -0.15$ T. Before each measurement the cooling fields were removed. The light is linearly polarized along the Y axis ($\mathbf{E}^\omega || Y$).

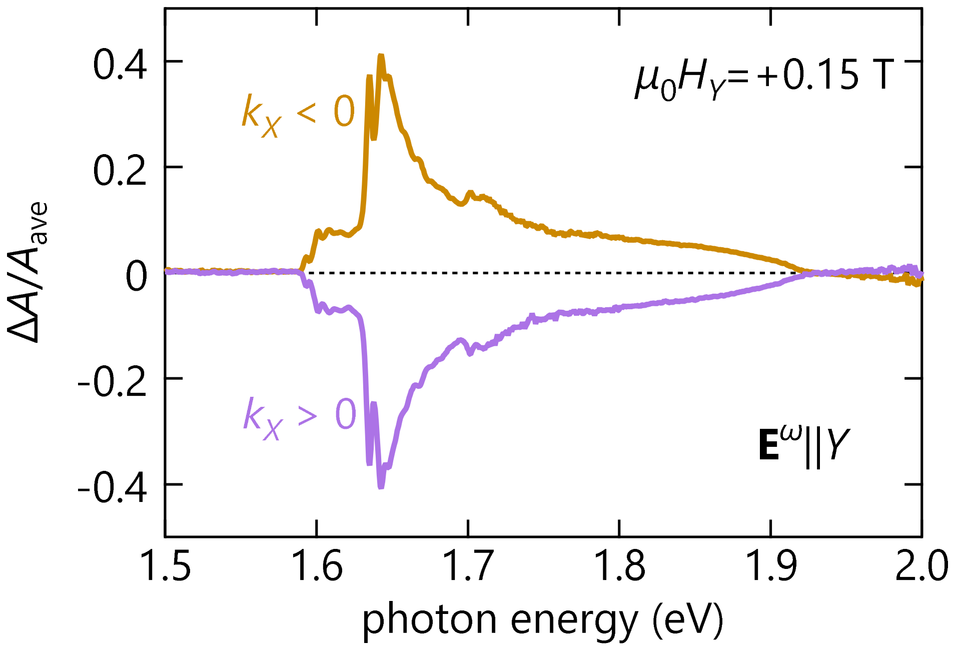

Supplementary Figure 6: Relative magnitude of nonreciprocal directional dichroism. Here, $\Delta A = A(+100\ \mathrm{kV\ m^{-1}}) - A(-100\ \mathrm{kV\ m^{-1}})$ and $A_{\mathrm{ave}} = [A(+100\ \mathrm{kV\ m^{-1}}) + A(-100\ \mathrm{kV\ m^{-1}})]/2$, where $A(+100\ \mathrm{kV\ m^{-1}})$ and $A(-100\ \mathrm{kV\ m^{-1}})$ corresponds to the spectra shown in Fig. 2a of the main text. The relative magnitude of the nonreciprocal directional absorption $\Delta A/A_{\mathrm{ave}}$ exceeds 0.4 (= 40%) at 1.65 eV.

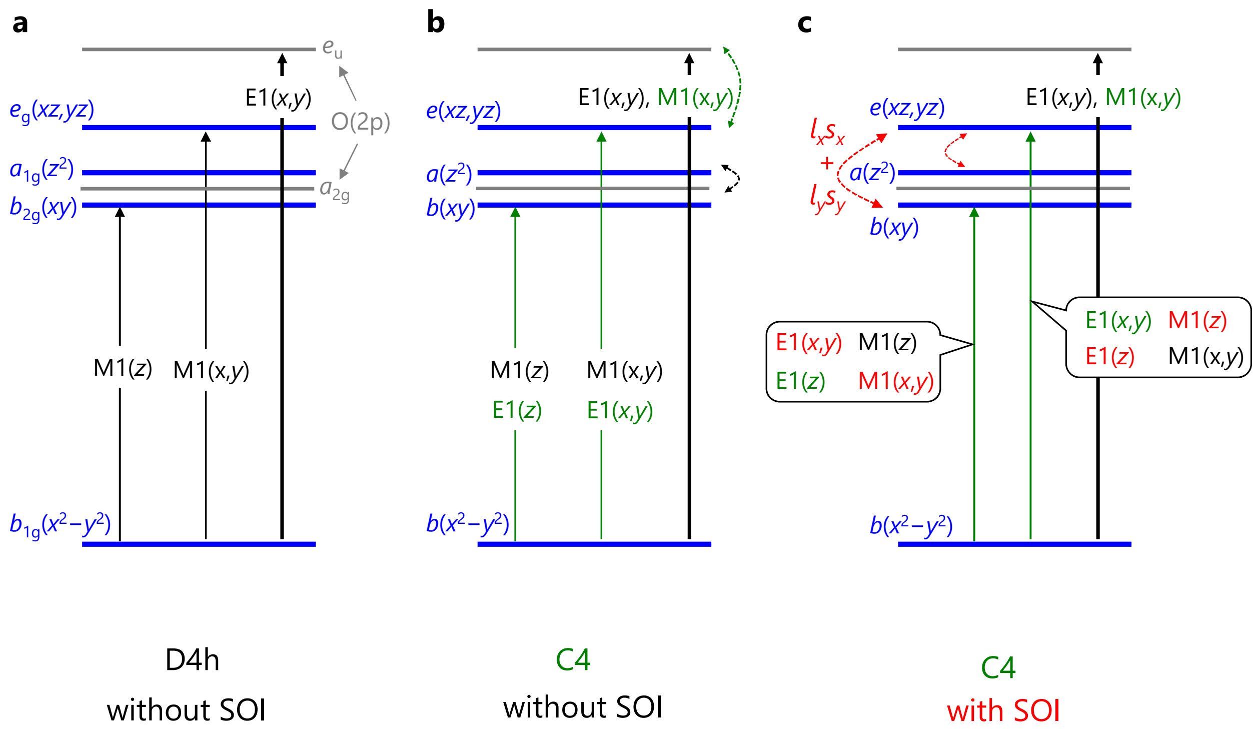

Supplementary Figure 7: Schematic electronic energy levels of a CuO_4^{6-} unit in Bi_2CuO_4. **a**, The energy level below 2 eV of a perfect square-planer CuO_4^{6-} unit with D4h symmetry, partly reproduced from Supplementary Ref. 3. The spin orbit interaction (SOI), $H_{SO} = \lambda(\mathbf{l}\cdot\mathbf{s}) = \lambda(l_x s_x + l_y s_y + l_z s_z)$, is not included. $b_{1g}(x^2\text{-}y^2)$, $b_{2g}(xy)$, $a_{1g}(z^2)$, $e_g(xz)$ and $e_g(yz)$ represent bonding molecular orbitals formed by Cu-3d and O-2p orbitals, which belong to the irreducible representations (IRs) of B_{1g}, B_{2g}, A_{1g}, E_g and E_g, respectively. (These orbitals consist of $d_{x^2-y^2}$, d_{xy}, d_{z^2}, d_{xz} and d_{yz} of the Cu ion, respectively). e_u is a pure oxygen bonding molecular orbital while a_{2g} is a pure oxygen nonbonding orbital. x, y and z denote the local coordinate axes with z being parallel to the crystallographic Z axis. **b**,**c** The energy level of a square-planer CuO_4^{6-} unit with actual C4 symmetry in Bi_2CuO_4, in the absence of SOI (**b**) and in the presence of SOI (**c**). Cu-3d and O-2p molecular orbitals are relabeled as $b(x^2\text{-}y^2)$, $b(xy)$, $a(z^2)$, $e(xz)$ and $e(yz)$ according to IRs of the C4 symmetry. Orbital hybridizations upon the symmetry lowering from D4h to C4 are represented by dashed allows in (b). Orbital hybridization due to the $l_x s_x + l_y s_y$ term is denoted by dashed allows in (c). Notations E1(i) [M1(i)] ($i = x,y,z$) mean the electric-dipole (magnetic-dipole) allowed transitions with an oscillating electric (magnetic) field of light along the i axis.

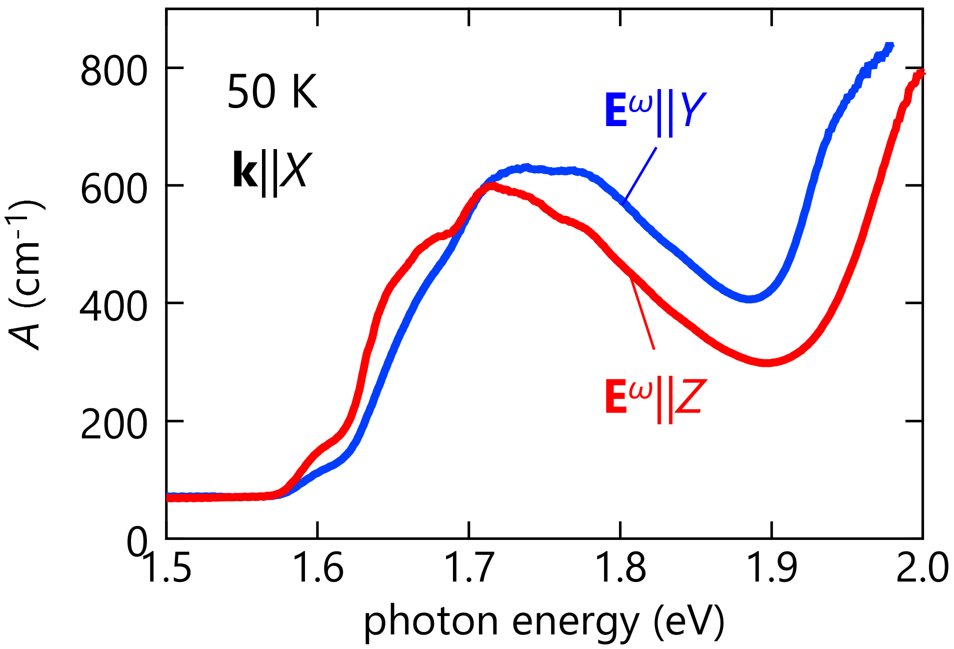

Supplementary Figure 8: Absorption spectra for the light polarization parallel to the *Y* and *Z* axes. The spectra were measured at 50 K in the paramagnetic phase.

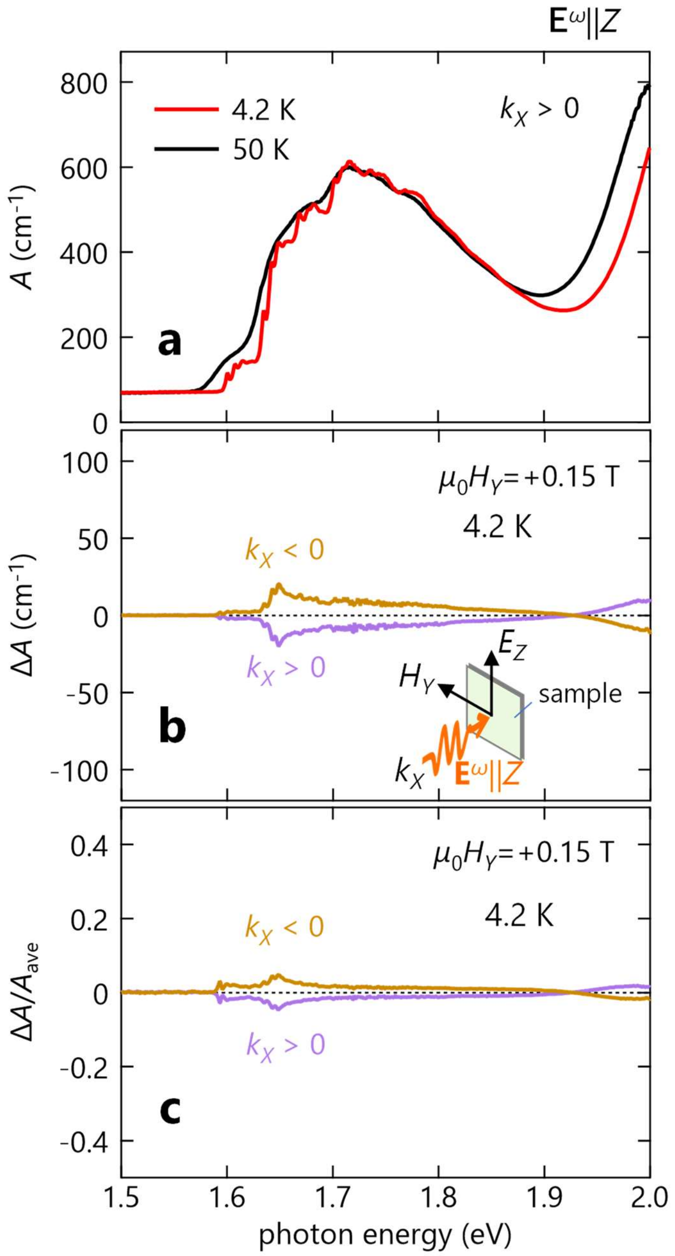

Supplementary Figure 9: Nonreciprocal directional dichroism for E$^{\omega}$||*Z*. **a**, The spectra of absorption coefficient (A) for $k_X > 0$ and **E**$^{\omega}$||Z measured at 0 T and at 4.2 K (red) and 50 K (black). The 4.2 K spectrum was measured after the sample was cooled with $\mu_0 H_Y$ = +0.15 T and E_Z = +100 kV m^{-1}. The cooling fields were removed before each measurement. **b**,**c**, The spectra of ΔA (**b**) and $\Delta A/A_{\mathrm{ave}}$ (**c**) for $k_X > 0$ (purple) and $k_X < 0$ (dark yellow). Here, ΔA = A(+100 kV m^{-1})–− A(−100 kV m^{-1}) and A_{ave} = [A(+100 kV m^{-1}) + A(−100 kV m^{-1})]/2, where A(+100 kV m^{-1}) and A(−100 kV m^{-1}) denote A measured after the sample was cooled with E_Z = +100 kV m^{-1} and −100 kV m^{-1}, respectively, and $\mu_0 H_Y$ = +0.15 T. The inset of (b) shows an experimental geometry.

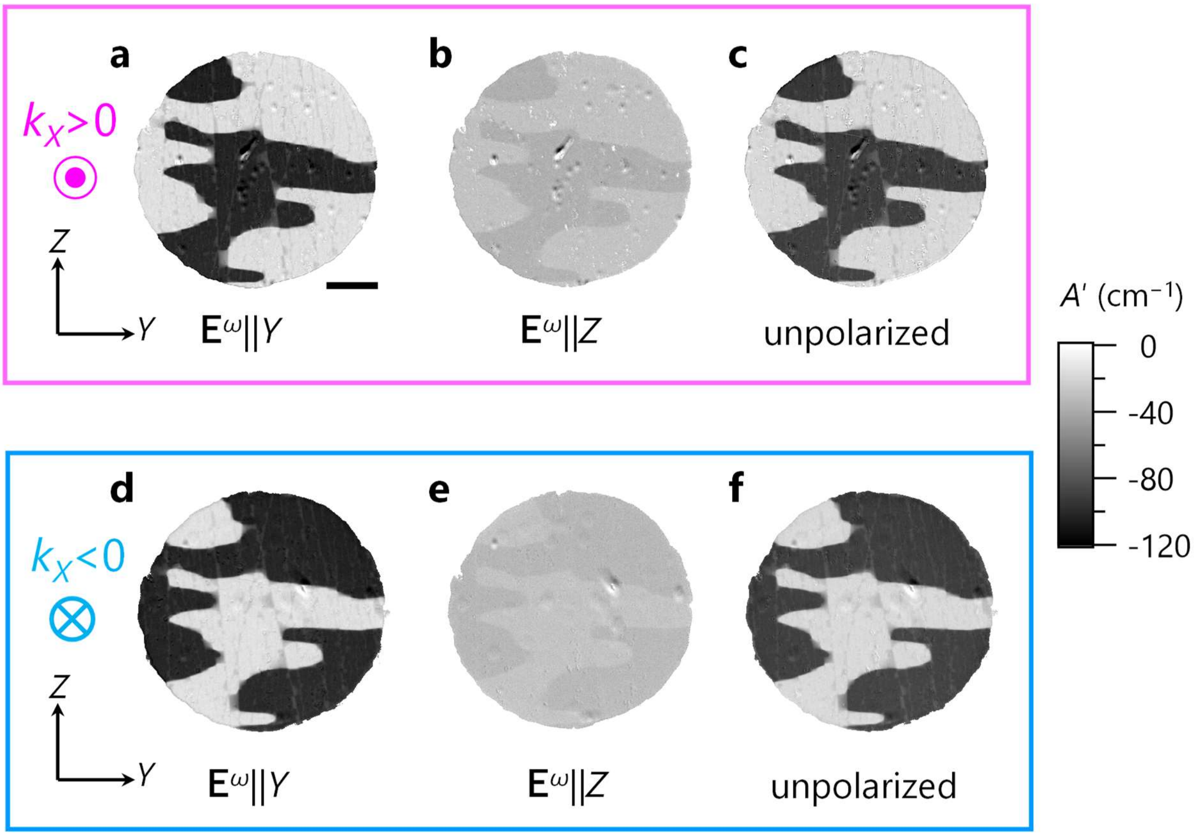

Supplementary Figure 10. Optical microscopy images obtained at various light conditions. **a-c,** A' images of an X-plane crystal at 5 K and 0 T after zero-field cooling for light linearly polarized along the Y axis ($\mathbf{E}^\omega||Y$) (**a**), the Z axis ($\mathbf{E}^\omega||Z$) (**b**), and unpolarized light (**c**). A' is the variation of absorption coefficient from 50 K. The propagation vector **k** of light is parallel to the +X axis ($k_X > 0$). **d**-**f**, A' images obtained in the same condition as (a)-(c), respectively, except that **k** is reversed ($k_X < 0$). A scale bar below (a) is 0.2 mm and applied to all the panels.

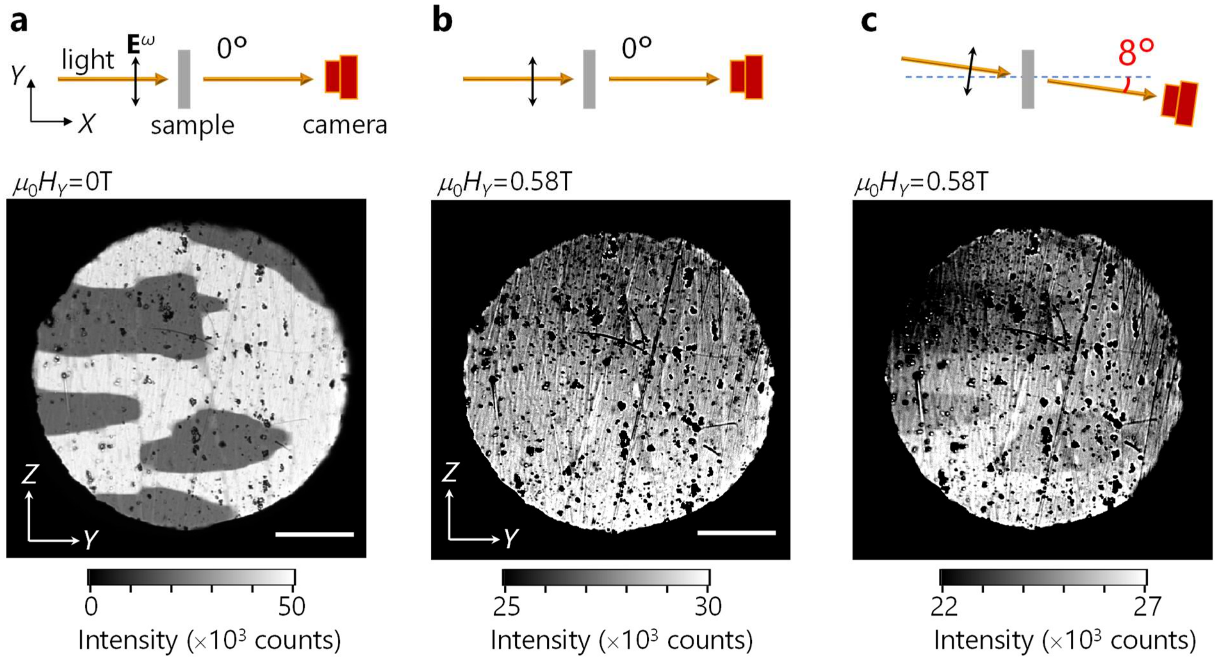

Supplementary Figure 11. Evidence for the existence of AFM domains in an applied magnetic field of 0.58 T. a-c, Optical microscopy images of an X-plane sample at 5 K after zero-field cooling. Note that the images correspond to intensity maps. The image in the panel (**a**) was measured in the absence of a magnetic field. A clear contrast corresponds to the L_{+Y} and L_{-Y} domains. As seen in (**b**), in an applied magnetic field of 0.58 T along the Y axis, AFM domains (L_{+X} and L_{-X}) are indistinguishable when the light propagation direction is parallel to the X axis. By contrast, as seen in (**c**), the L_{+X} and L_{-X} domains are clearly visible when the light propagation direction is slightly tilted from the X axis toward the Y axis by ~8°. The pattern of the L_{+X} and L_{-X} domains in (c) is approximately the same as that of the L_{+Y} and L_{-Y} domains shown in (a). Scale bars below (a) and (b) are 0.2 mm.

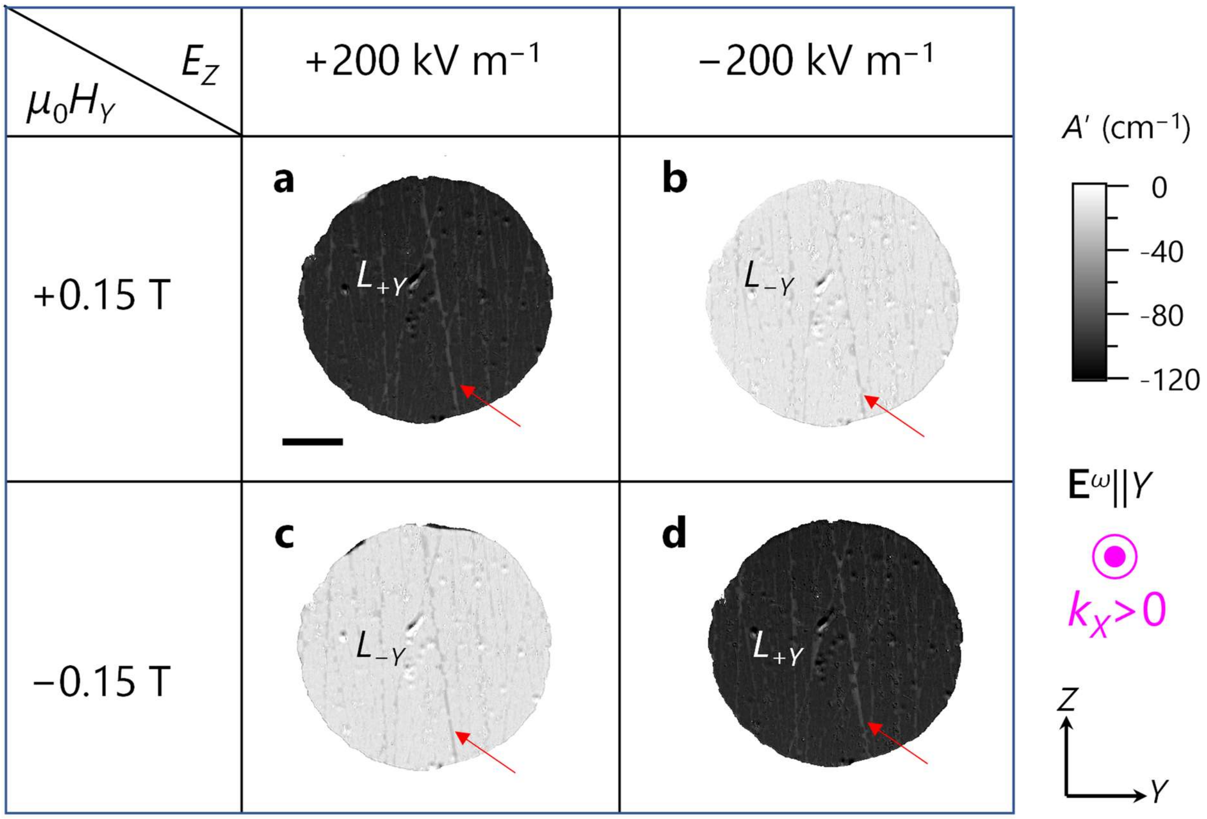

Supplementary Figure 12. Optical microscopy images after a magnetoelectric cooling procedure. a-d, A' images of an X-plane crystal for $k_X > 0$ and $\mathbf{E}^{\omega}||Y$ at 5 K after a magnetoelectric cooling with four combinations of signs of cooling magnetic ($\mu_0 H_Y$) and electric (E_Y) fields; (+,+) (**a**), (+,−) (**b**), (−,+) (**c**), and (−,−) (**d**). A' is the variation of absorption coefficient from 50 K. The magnitudes of $\mu_0 H_Y$ and E_Y are 0.15 T and 200 kV m^{-1}, respectively. The cooling fields were removed before each microscope observation. Nearly single domain states of L_{+Y} (dark) are achieved for (+,+) and (−,−) while those of L_{-Y} (bright) for (+,−) and (−,+). However, stripe-shaped regions (pointed by red arrows) have a slightly weaker contrast than other regions. A scale bar in (a) is 0.2 mm and applied to all the panels.

Supplementary Note 1: Microscopic magnetic toroidal moment in Bi_2CuO_4

In this section, we calculate the microscopic magnetic toroidal moment **t** of Bi_2CuO_4 according to a commonly used definition $\mathbf{t} \propto \sum_{n=1-4} \mathbf{r}_n \times \mathbf{S}_n$, where $\mathbf{r}_n$ is the position vector and $\mathbf{S}_n$ is the spin of the *n*-th magnetic ion in a unit cell (Supplementary Ref. 4). In Bi_2CuO_4, there are four Cu ions in the primitive unit cell (i.e., n = 1-4), which are labelled as shown in Supplementary Figs. 1a,1b. The fractional coordinates in the (a, b, c) frame are (1/4, 1/4, z_0) for Cu1, (1/4, 1/4, z_0−1/2) for Cu2, (3/4, 3/4, −z_0+3/2) for Cu3, and (3/4, 3/4, −z_0+1) for Cu4, with z_0 = 0.921 (Supplementary Ref. 5). This yields a relation $\mathbf{r}_1 - \mathbf{r}_3 = \mathbf{r}_2 - \mathbf{r}_4$. In the collinear AFM phase, $\mathbf{S}_1 = \mathbf{S}_2 = -\mathbf{S}_3 = -\mathbf{S}_4$. Using these relations, **t** is rewritten as $\mathbf{t} \propto \mathbf{r}_{13} \times \mathbf{S}_1$, where $\mathbf{r}_{13} \equiv \mathbf{r}_1 - \mathbf{r}_3$.

As an example, let us calculate **t** of the L_{+X} domain by considering $\mathbf{S}_1 = (S,S,0)/\sqrt{2}$ (i.e., $\mathbf{S}_1 \parallel +X$). Here, S is the magnitude of **S**. We note that $\mathbf{r}_{13}$ takes four distinct directions depending on the choice of the unit cell, as denoted by sky-blue arrows in Supplementary Figs, 1c-1f. They are explicitly expressed as $\mathbf{r}_{13}$ = (a/2, a/2, −0.342c) for Supplementary Fig. 1c, $\mathbf{r}_{13}$ = (−a/2, −a/2, −0.342c) for Supplementary Fig. 1d, $\mathbf{r}_{13}$ = (a/2, −a/2, −0.342c) for Supplementary Fig. 1e, and $\mathbf{r}_{13}$ = (−a/2, a/2, −0.342c) for Supplementary Fig. 1f. The corresponding calculated values of **t** are $\mathbf{t} \propto (3.42cS, -3.42cS, 0)/\sqrt{2}$, $\mathbf{t} \propto (3.42cS, -3.42cS, 0)/\sqrt{2}$, $\mathbf{t} \propto (3.42cS, -3.42cS, aS)/\sqrt{2}$, and $\mathbf{t} \propto (3.42cS, -3.42cS, -aS)/\sqrt{2}$, respectively. In all the case, **t** has the ab-plane (XY-plane) component that is independent of the choice of the unit cell. This XY-plane component of **t** is parallel to the −Y axis, which is consistent with the fact that the *mm'm* magnetic point group of the L_{+X} domain state supports magnetic toroidal moment **T** along the Y axis. In disagreement with the *mm'm* group, by contrast, **t** also has the c-axis (Z-axis) component with opposite signs for the unit cells shown in Supplementary Figs. 1e and 1f. However, there is no Z-axis component that is independent of the unit-cell choice. This suggests that the appearance of finite Z-axis component of **t** is artifact and physically meaningless.

As a way to obtain a unit-cell independent value of the microscopic magnetic toroidal moment, we propose to use a $\sqrt{2} \times \sqrt{2}$ unit cell, as shown in Supplementary Fig. 1g, which is doubled in size compared to the primitive unit cell and thus contains eight Cu ions. We express a toroidal moment of this new unit cell as $\mathbf{t}' \propto \sum_{n=1-8} \mathbf{r}_n \times \mathbf{S}_n$. Note that Cu5, Cu6, Cu7 and Cu8 are equivalent to Cu1, Cu2, Cu3 and Cu4, respectively, in terms of the translational symmetry. According to the simple calculation, one obtains $\mathbf{t}' \propto (\mathbf{r}_{13} + \mathbf{r}_{57}) \times \mathbf{S}_1$ with $\mathbf{r}_{57} \equiv \mathbf{r}_5 - \mathbf{r}_7$.

Importantly, $\mathbf{r}_{13} + \mathbf{r}_{57} = (0, 0, -0.648c)$ has only the negative Z axis component. As a result, $\mathbf{t}'$ is parallel to the $-Y$ axis (since $\mathbf{S}_1 \parallel +X$), in agreement with the $mm'm$ magnetic point group. The directions of $\mathbf{t}'$ for the other three domains are readily obtained: $\mathbf{t}' \parallel +Y$ for the L_{-X} domain ($\mathbf{S}_1 \parallel -X$), $\mathbf{t}' \parallel +X$ for the L_{+Y} domain ($\mathbf{S}_1 \parallel +Y$), and $\mathbf{t}' \parallel -X$ for the L_{-Y} domain ($\mathbf{S}_1 \parallel -Y$). This conclusion is general as long as the $\sqrt{2} \times \sqrt{2}$ unit cell is selected.

Supplementary Note 2: Characteristics of NDD in Bi_2CuO_4

In this section, we compare the nonreciprocal directional dichroism (NDD) in the present antiferromagnetic (AFM) material Bi_2CuO_4 and a weak ferromagnet CuB_2O_4 (Supplementary Ref. 6), both of which have square-planar CuO_4 units. As mentioned in the main text, the large NDD (for $\mathbf{E}^{\omega} \| Y$ and $\mathbf{H}^{\omega} \| Z$) in Bi_2CuO_4 arises likely due to the weak inversion symmetry breaking at the Cu site, because this can make the E1 transition weak and comparable in magnitude to the M1 transition, thus enhancing the E1-M1 interference effect. This scenario is essentially the same as that proposed for the gigantic NDD observed in CuB_2O_4 (Supplementary Ref. 6). We note that the relative magnitude of NDD for Bi_2CuO_4 (~40%) is weaker than that for CuB_2O_4 (more than 100%). It indicates that the balance of the E1 and M1 transition amplitudes is less optimized in Bi_2CuO_4 compared to CuB_2O_4. This might be partly ascribed to the stronger $3d$-$2p$ covalency in Bi_2CuO_4, as it dilutes (or weakens) the $3d$ character of the electronic levels responsible for the NDD. We also note that there is another qualitative difference between the two materials; that is, the site symmetry at the Cu ion is S4 in CuB_2O_4 but C4 in Bi_2CuO_4. In Bi_2CuO_4, the E1 (M1) transition from the ground state $b(x^2\text{-}y^2)$ to the SOI-modulated excited state $b(xy)$, which is located at around 1.6-1.7 eV, is allowed not only with $\mathbf{E}^{\omega} \| Y$ ($\mathbf{H}^{\omega} \| Z$) but also with $\mathbf{E}^{\omega} \| Z$ ($\mathbf{H}^{\omega} \| Y$) (see Supplementary Fig. 7c). As a result, NDD for light propagating along the X axis is expected to occur not only for $\mathbf{E}^{\omega} \| Y$ but also for $\mathbf{E}^{\omega} \| Z$. Such a NDD signal is indeed observed, as shown in Supplementary Fig. 9. By contrast, a corresponding E1 ($\mathbf{E}^{\omega} \| Z$) transition is forbidden in the S4 symmetry. This is consistent with the absence of a NDD signal in CuB_2O_4 for this light polarization direction (Supplementary Ref. 6).

Supplementary Note 3: Evidence for the existence of the AFM order in a magnetic field of 0.58 T

In an applied magnetic field of 0.58 T along the *Y* axis ($\mu_0 H_Y = 0.58$ T), the L_{+X} and L_{-X} states are expected to be stabilized. When we use incident light parallel to the *X* axis (**k** || *X*) in an optical microscopy imaging, a uniform optical image of a sample is obtained, as displayed in Fig. 5c of the main text. This is because A_{NDD} is zero for both the L_{+X} and L_{-X} states [see Eq. (2) of the main text]. However, one may consider that the uniform image is insufficient to prove the existence of the L_{+X} and L_{-X} states; for example, the uniform image could also be produced if the AFM order was destroyed by a magnetic field. To exclude such a possibility, we performed an optical microscopy imaging of the *X*-plane sample (the same one used in the main text) in an applied $\mu_0 H_Y = 0.58$ T, using incident light slightly tilted from the *X* axis toward the *Y* axis. With this tilting, **k** acquires a component normal to **L**, which yields two distinct values of A_{NDD} for the L_{+X} and L_{-X} states. As a result, the sample image should show a contrast if the domains are present. The schematic of an experimental setup and the results are shown in Supplementary Fig. 11. Supplementary Figure 11c clearly shows that the contrast of the image appears with the oblique light. This provides direct evidence for the existence of the L_{+X} and L_{-X} domains in $\mu_0 H_Y = 0.58$ T. We also note that the domain pattern in $\mu_0 H_Y = 0.58$ T is approximately the same as that in 0 T. This strongly suggests that the domain transformation is dominated by a Néel vector rotation within each domain.

Supplementary References

1. Zhao, L. *et al.* Magnetically induced ferroelectricity in Bi_2CuO_4. *Phys. Rev. B* **96**, 054424 (2017).
2. Yuan, B. *et al.* Neutron scattering study of magnetic anisotropy in a tetragonal antiferromagnet Bi_2CuO_4. *Phys. Rev. B* **103**, 134436 (2019).
3. Pisarev, R. V., Pavlov, V. V., Kalashnikova, A. M. & Moskvin, A. S. Near-band gap electronic structure of the tetragonal rare-earth cuprates R_2CuO_4 and the bismuth cuprate Bi_2CuO_4. Phys. Rev. B **82**, 224502 (2010).
4. Schmid, H. On ferrotoroidics and electrotoroidic, magnetotoroidic and piezotoroidic effects. *Ferroelectrics* **252**, 41–50 (2001).
5. Yamada, K. *et al.* Three-dimensional antiferromagnetic order and anisotropic magnetic properties in Bi_2CuO_4. *J. Phys. Soc. Jpn.* **60**, 2406-2414 (1991).

6. Saito, M., Taniguchi, K. & Arima, T. Gigantic optical magnetoelectric effect in CuB_2O_4. *J. Phys. Soc. Jpn.* **77**, 013705 (2008).
