# Supplementary material for: Visualizing rotation and reversal of the Néel vector through antiferromagnetic trichroism
Source: Nat Commun. 2022 Feb 4;13:697. doi: 10.1038/s41467-022-28215-w (PMC8816959; doi:10.1038/s41467-022-28215-w)
Supplement: Supplementary file 2 — Description of Additional Supplementary Files [file 41467_2022_28215_MOESM2_ESM.pdf]

Title: Supplemental Movie 1
Description: A complete set of sample images at 5 K in varied magnetic fields. The images correspond to the variation of absorption coefficient from 50 K.

Title: Supplemental Movie 2
Description: Real-time video of the sample at 5 K in varied magnetic fields. The brightness of the video corresponds to the transmitted light intensity.
